# Electrostatic Surface Properties of Blood and Semen Extracellular Vesicles: Implications of Sialylation and HIV-Induced Changes on EV Internalization

**DOI:** 10.3390/v12101117

**Published:** 2020-10-01

**Authors:** Hussein Kaddour, Tyler D. Panzner, Jennifer L. Welch, Nadia Shouman, Mahesh Mohan, Jack T. Stapleton, Chioma M. Okeoma

**Affiliations:** 1 Department of Pharmacology, Stony Brook University Renaissance School of Medicine, Stony Brook, NY 11794, USA; hussein.kaddour@stonybrook.edu (H.K.); tyler.panzner@stonybrook.edu (T.D.P.); nadia.shouman@stonybrook.edu (N.S.); 2 Department of Microbiology and Immunology, Carver College of Medicine, University of Iowa, Iowa City, IA 52242, USA; jennifer-welch@uiowa.edu (J.L.W.); jack-stapleton@uiowa.edu (J.T.S.); 3 Medical Service, Iowa City Veterans Affairs Medical Center, Iowa City, IA 52246, USA; 4 Department of Internal Medicine, Carver College of Medicine, University of Iowa, Iowa City, IA 52242, USA; 5 Host Pathogen Interaction Program, Southwest National Primate Research Center, Texas Biomedical Research Institute, San Antonio, TX 78227, USA; mmohan@txbiomed.org

**Keywords:** HIV-1, blood, semen, extracellular vesicles, zeta potential, biological membranes, glycocalyx

## Abstract

Although extracellular vesicle (EV) surface electrostatic properties (measured as zeta potential, ζ-potential) have been reported by many investigators, the biophysical implications of charge and EV origin remains uncertain. Here, we compared the ζ-potential of human blood EVs (BEVs) and semen EVs (SEVs) from 26 donors that were HIV-infected (HIV+, *n* = 13) or HIV uninfected (HIV-, *n* = 13). We found that, compared to BEVs that bear neutral surface charge, SEVs were significantly more negatively charged, even when BEVs and SEVs were from the same individual. Comparison of BEVs and SEVs from HIV- and HIV+ groups revealed subtle HIV-induced alteration in the ζ-potential of EVs, with the effect being more significant in SEVs (∆ζ-potential = −8.82 mV, *p*-value = 0.0062) than BEVs (∆ζ-potential = −1.4 mV, *p*-value = 0.0462). These observations were validated by differences in the isoelectric point (IEP) of EVs, which was in the order of HIV + SEV ≤ HIV-SEV ≪ HIV + BEV ≤ HIV-BEV. Functionally, the rate and efficiency of SEV internalization by the human cervical epithelial cell line, primary peripheral blood lymphocytes, and primary blood-derived monocytes were significantly higher than those of BEVs. Mechanistically, removal of sialic acids from the surface of EVs using neuraminidase treatment significantly decreased SEV’s surface charge, concomitant with a substantial reduction in SEV’s internalization. The neuraminidase effect was independent of HIV infection and insignificant for BEVs. Finally, these results were corroborated by enrichment of glycoproteins in SEVs versus BEVs. Taken together, these findings uncover fundamental tissue-specific differences in surface electrostatic properties of EVs and highlight the critical role of surface charge in EV/target cell interactions.

## 1. Introduction

Cell membrane surface charge is the net sum of the charges of its components and determines the electrostatic surface properties of a cell. Phospholipids mainly contribute to this charge, and while phosphatidylcholine (PC) is neutral at physiological pH, phosphoserine (PS) and phosphoinositol (PI) confer a net negative charge to cell membranes [1]. Membrane proteins, glycoproteins, proteoglycans, and nucleic acids also contribute to the membrane surface charge. Cell membrane surface charge is dynamic, and its spatiotemporal variation strongly depends on the physiological state of the cell. Cancer cells, for instance, are more negatively charged than normal cells [2]. The membrane surface charge of biomolecules for cargo delivery and of recipient cells plays a role in the initial homotypic or heterotypic electrostatic interactions that precede target recognition, binding, and internalization of the delivery particle into the recipient cells. For instance, phagocytic cells preferentially internalize negatively charged nanoparticles whereas non-phagocytic cells favor positively charged particles [3]. The membrane surface charge of cells also regulates intracellular signaling. For example, K-ras, c-Src, and Rac1 signaling participate in surface charge-directed signaling [4]. Cellular membrane components as well as their intermolecular interactions determine cellular membrane net charge, which can be measured as ζ-potential [5]. Thus, measurement of ζ-potential is a reliable way to detect molecular changes that have occurred within cells.

Another measure to detect molecular changes in live cells is to examine the physical and compositional properties of the cell’s extracellular milieu, including extracellular vesicles (EVs). While there are two main subtypes of EVs (exosomes and microvesicles) in body fluids, they exhibit many similarities in both their physical and compositional properties. Thus, we use the term EVs, which encompasses all subtypes, in this study. EVs are mediators of distal and proximal cellular communication, as well as activators or inhibitors of a wide range of cellular processes, depending on the biological context. For instance, EVs play roles in viral defense and viral spreading [6,7,8], cancer metastasis and treatment [9,10], as well as chemoresistance and drug delivery [11,12]. As in cells, the electrochemical property of the EV surfaces also depends on the outer membrane cargo, composed of an intermolecular network of lipids [13], proteins [14], glycans [15], and possibly nucleic acids [16]. While EV surface charge has scarcely been reported as ζ-potential values [17,18,19,20], ζ-potential alone in the absence of pH at which measurements were taken is insufficient to infer EV surface charge status. This is because pH variation drastically affects the surface charge [21]. The isoelectric point (IEP), which is the pH at which the net charge is zero (also known as point of zero charge (PZC)), is a better parameter to assess EV surface charge. In practical terms, EV surface charge may be used to evaluate the adhesion forces between EVs and a target substrate allowing prediction of the likelihood of EV attachment to a surface or internalization by target cells. Thus, surface charge may be used to design EV therapeutics based on electrostatic adsorption.

Sialylation, which is the covalent addition of negatively charged sialic acid residues to the terminal ends of N and O-linked glycoproteins, and glycosphingolipids [22,23], is a major determinant of cell and EV membrane surface charge. In addition to decreasing overall membrane ζ-potentials, sialic acids serve as ligands for a variety of cellular receptors [24] and play key roles in numerous disease states, including viral pathogenesis and cancer [25,26,27,28,29,30]. Previous studies that investigated the role of sialylation and overall ζ-potential in EV internalization by recipient cells showed contradictory results, where sialylation was found to either facilitate or hinder EV internalization [31,32,33,34,35]. However, these studies utilized EVs that were primarily derived from cultured cells [31,32,33,34,35]. In the context of human diseases, and to the best of our knowledge, there has been no study assessing the effect of a specific disease condition, such as HIV infection on body fluid EV ζ-potential, sialylation, or cellular uptake. Although the biological characteristics and functions of EVs are becoming increasingly clear, major physical properties such as membrane surface charge, which may contribute to the behavior and function of EVs, remain unexplored. This is especially important because EVs carry markers of the producer cells. As a result, if the producer cells are healthy or pathologic, EVs will carry markers corresponding to the state of the cell [7,36]. In this case, the functions of EVs depend on their cargo, which they derive from the producer cells. A good example of this producer cell mediated regulation of EV composition and function is the differences between autologous blood and semen derived EVs in HIV inhibition [37,38,39] and proteome composition [38]. In the context of HIV, blood and semen EVs (BEVs and SEVs) are of particular importance since both body fluids play important roles in HIV transmission.

In this study, we sought to further understand the differences between blood and semen derived EVs by (i) providing thorough assessment of the surface electrostatic charge of BEVs and SEVs, (ii) determining the effect of HIV on EV surface charge, and (iii) evaluating if differences in surface charge affect EV internalization. We found that amongst autologous EV, BEVs bear neutral while SEVs bear negative charge. BEVs and SEVs from HIV+ donors were, respectively, slightly or significantly more negatively charged than their HIV- counterparts. We also found that EV internalization rate and efficiency positively correlate with EV negative surface charge. Furthermore, neuraminidase digestion, which removes terminal sialic acids, dampened the net negative charge on SEVs surface and impaired their cellular internalization. Conversely, BEV surface charge and internalization were largely unaffected by desialylation.

## 2. Materials and Methods

### 2.1. Ethical Approvals

The Institutional Review Board (IRB) of the University of Iowa and Stony Brook University approved the use of human specimens (IRB numbers 201608703 and 1106876, respectively). HIV- and HIV+ subjects consented to participate in this study via written informed consent. All samples were received unlinked to any identifiers. All experiments were performed in accordance with the approved university guidelines and regulations.

### 2.2. Participants

The clinical and demographic characteristics of HIV+ (*n* = 13) and HIV- (*n* = 3) donors of matched blood and semen used in this study were previously published [38]. HIV- donors had no history of HIV, hepatitis B virus (HBV), and hepatitis C virus (HCV) infection at time of participation. HIV+ donors were ART-suppressed with viral load <50 copies/mL at the time of specimen donation [38]. 

### 2.3. Cells

TZM-bl cells were obtained through the NIH AIDS Reagent Program and maintained in Dulbecco’s modified Eagle’s medium (DMEM) (Gibco-BRL/Life Technologies) supplemented with 10% fetal bovine serum (FBS) (Atlanta Biologicals, Flowery Branch, GA, USA) that was EV-depleted by 16 h ultracentrifugation at 100,000× *g*, 1% Penicillin-streptomycin (Thermofisher, Grand Island, NY, USA), 1 µg/mL Amphotericin B (Thermofisher), 2 mM sodium pyruvate (Corning, Corning, NY, USA), 1% of glutamate (Thermofisher), and 10 mM HEPES buffer (Fisher Biotech, Fair Lawn, NJ, USA) at pH 8. Isolation of primary cells was performed as previously described [37,39,40]. Frozen primary peripheral blood mononuclear cells (PBMCs), isolated from blood collected from 3 healthy donors, were thawed at 37 °C and individually washed with complete Roswell Park Memorial Institute (RPMI) media (Corning). Cell viability was determined by the trypan blue method to be >90%. Cells were plated in 6-well plate (Cyto-One, USA Scientific) at 3 × 10^6^/mL, and allowed to adhere in a 5% CO_2_ incubator at 37 °C for 2 h. Non-adherent cells were collected as Peripheral blood lymphocytes (PBLs) and cultured in a separate plate. Adherent cells were carefully washed with RPMI and cultured as primary monocytes.

### 2.4. EV Purification from Human Semen and Human Blood

The isolation and characterization of the EVs used in this study was recently described [38]. Briefly, blood or seminal plasma were centrifuged at 2000× *g* for 10 min and 10,000× *g* for 30 min, to pellet cellular debris. ExoQuick (EQ, System Biosciences, Palo Alto, CA, USA) was added at a ratio of 4:1 (plasma: EQ) and incubated at 4 °C overnight. The mixture was then centrifuged at 1500× *g* for 30 min at 4 °C and the EQ/EV-free seminal plasma was removed. Residual EQ was removed by centrifuging the EV pellet at 1500× *g* for 5 min and discarding the supernatant. The EV pellet was re-suspended in PBS in 1/10 of the original volume. Protein quantification was determined by NanoDrop absorbance at 280 nm, and samples were frozen at −80 °C until use.

### 2.5. ζ-Potential Measurements

ζ-potential measurements were performed using ZetaView PMX 110 (Particle Metrix, Mebane, NC) and corresponding software (version 8.04.02). EVs were diluted in ultrapure water (1:20,000–1:320,000) to reach to a particle number per frame of 50 to 300, ideal for Nano Tracking Analysis (NTA). Temperature was set at 25 °C and 5 cycles at high sensitivity setting were performed per measurement for a total of three to ten measurements per sample. As a control, 10 measurements were collected for representative samples of each group to test sample stability during measurements. Camera settings for all measurements were fixed at a sensitivity and a shutter of 91 and 70, respectively. The high sensitivity settings were recommended by the manufacturer to capture the most EVs.

### 2.6. pH Dependence

In total, 2 µL of pooled (*n* = 10) HIV- or HIV+, BEV or SEV, diluted 1:20,000 in 40 mL H_2_O, were titrated with 10 µL NaOH (10 mM) at a time. The pH was recorded, and the ζ-potential was measured in quintuplets.

### 2.7. EV Labelling

In total, 300 µg pooled (*n* = 10) HIV- or HIV+, BEV or SEV were diluted with 1× PBS to 499 µL, to which 1 µL ExoGlow protein labelling dye (System Biosciences) was added, and the mixture was incubated at 37 °C for 20 min with constant shaking (1000 rpm). A 1x PBS solution was used as a negative control. Labelled EVs were precipitated by 167 µL EQ TC (System Biosciences) for 2 h at 4 °C and re-concentrated by centrifugation at 10,000 g for 10 min. Labelled EV pellets were dissolved in 1x PBS and their particle concentration was measured by NTA.

### 2.8. EV Internalization

TZM-bl cells were seeded overnight in a 96-well plate (Cyto-One, USA Scientific) at 10,000 cells per well. ExoGlow labelled EVs (100 µg/mL, corresponding to ~2–3 × 10^10^ particles/mL) were diluted in complete DMEM to which 20 µL/mL NucBlue Live ReadyProbes (EasyProbes, Thermofisher) was added. Kinetic imaging of live cells was performed using LionHeart (BioTek, Winooski, VT, USA) with a 10× objective and images of 4 adjacent fields of view were taken. Images were stitched and fluorescence in the Green-fluorescence protein (GFP) channel was measured within a secondary mask that was set at 6 µm expanding from a primary mask, which was set in the DAPI channel and reduced by 4 µm. This quantification strategy reduced the background signal from non-internalized EVs and accounted for any potential differential cell growth. Subsequently, cells’ background was subtracted, and fluorescence values were normalized relative to labelled PBS control. The luciferase assay post-internalization was performed according to a published procedure [41].

PBLs and monocytes were activated overnight by plate-bound anti-CD3 (10 µg/mL) and soluble anti-CD28.8 (2 µg/mL) (BioLegend, San Diego, CA, USA). To activated PBLs and monocytes, ExoGlow labelled HIV- BEV or SEV, or labelled vehicle control, were added at 100 µg/mL (~2–3 × 10^10^ particles/mL) and kinetic imaging of live cells were recorded every three hours for 24 h. Total green fluorescence was measured, cells background was subtracted, and values were normalized to labelled PBS control. The MTT viability assay post-internalization was performed according to published procedure [41].

### 2.9. α-Neuraminidase Treatment

In total, 100 µg of pooled (*n* = 10) HIV- or HIV+, BEV or SEV was mixed with 250 units of α2-3,6,8 Neuraminidase (New England BioLabs, Ipswich, MA), or equivalent volume of PBS control, in 100 µL GlycoBuffer1 (5 mM CaCl_2_, 50 mM sodium acetate, pH 5.5 at 25 °C) and the reaction was incubated at 37 °C for 1 h. Samples were then inactivated at 65 °C for 10 min, according to the manufacturer’s instructions, before measurement of ζ-potential, EV labelling, and internalization assessment.

### 2.10. Statistics

**Figure 5:**
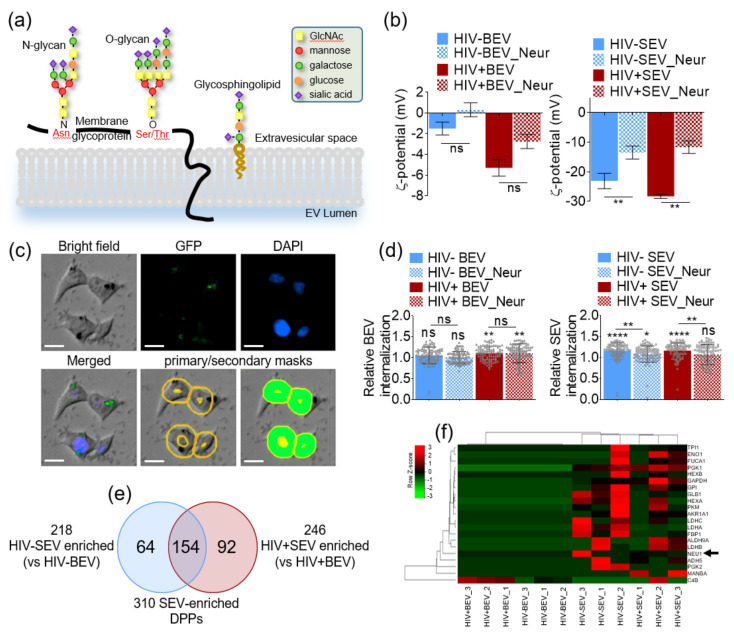
Sialic acids affect SEV ζ-potential and internalization efficiency. (**a**) Schematic of EV surface membrane showing a membrane glycoprotein and glycosphingolipid decorated with sialic acids. (**b**) ζ-potential of HIV- (blue) and HIV+ (red) BEVs and SEVs before (full bars) and after (pattern bars) neuraminidase treatment. (**c**) Representative 10× images of EV internalization. Scale bar is 20 µm. Primary and secondary masks were set in the DAPI and GFP channels, respectively. Min and Max object size for the primary mask were 5 and 55 µm, respectively. GFP intensity for each cell was measured by reducing the primary mask with 4 µm and expanding the secondary mask with 8 µm. (**d**) Relative EV internalization at 24 h of intact and neuraminidase-treated HIV- and HIV+ BEVs and SEVs. Represented data are an average of 16 fields of view per well and 6 wells per treatment. (**e**) Venn diagram between the differentially present proteins (DPPs) enriched in SEVs as compared to BEV from the HIV- and HIV+ groups, as analyzed in [51]. (**f**) Heatmap clustering analysis of the 21 glycocalyx-related SEV-enriched DPPs (rows). Columns correspond to the analyzed EV samples. Spearman rank correlation single linkage was applied to rows and columns. Heatmap was generated using heatmapper, an online data visualization application [53]. Ordinary one-way ANOVA test was performed to determine the differences between the treatments and the labelled PBS. Unpaired t-test with Welch’s correction was used to determine the differences between the two-group comparisons. Error bars indicate S.D. * *p* < 0.05; ** *p* < 0.01; **** *p* < 0.0001; ns, non-significant.

Graphpad Prism (v 8.4.2) was used to plot all the graphs and to determine the statistical significance. For a two-group comparison, an unpaired t-test with Welch’s correction was used to determine the differences between the groups (Figures 2c,e,f, 5b,d and S1a,c,d), unless all samples were matched in which case a paired and parametric t-test was employed (Figures 2b and S1b). For multiple comparisons, ordinary one-way ANOVA test with Dunnett’s correction was used to determine the differences between the groups as compared to the control (Figures 3d, 4c,d and 5d). For 2-variable multiple comparisons, two-way ANOVA with Sidak’s correction was used and the predicted mean difference with adjusted *p*-value reported (Figure 4e). Error bars represent either standard deviation (S.D.) across multiple measurements, or standard error of the mean (S.E.M.) across independent experiments.

## 3. Results

### 3.1. ζ-Potential of BEV and SEV Is Stable and Time- and Concentration- Independent

**Figure 1:**
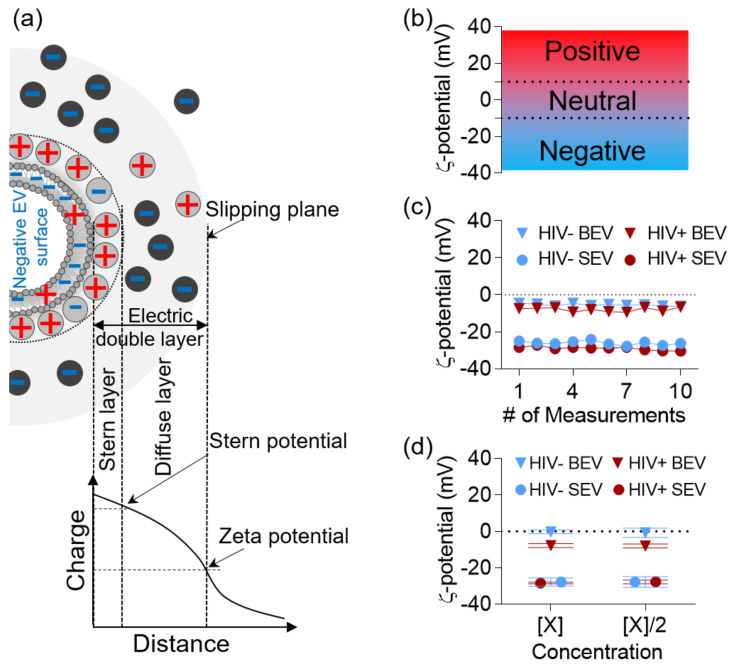
ζ-potential measurements using Nano Tracking Analysis (NTA). (**a**) Schematic showing that the ζ-potential corresponds to the electrokinetic potential at the slipping plane between the diffuse layer of the vesicle and the diluent. (**b**) Graph showing ζ-potential ranges of EVs. (**c**) Graph showing 10 repeat measurements of the same sample (pooled, *n* = 10, HIV- or HIV+, BEV or SEV). (**d**) Graph of ζ-potential measurements of the same sample before and after dilution.

ζ-potential corresponds to the electric potential near the surface of a charged particle [42] (Figure 1A), and represents the charge of the particle in relation to its surrounding milieu. ζ-potential is measured in ranges where values between −10 to +10 mV are considered neutral while values > +30 mV or < −30 mV are considered strongly cationic or strongly anionic, respectively [21] (Figure 1B). ζ-potential of EVs can be measured using Nanoparticle Tracking Analysis (NTA) [43]. NTA, in general, requires diluted samples, in the range of 10^7^ to 10^9^ particles/mL [44], which corresponds to ~0.1 or 10 µg protein/mL in the case of BEVs and SEVs, respectively. Therefore, purified and pooled BEVs and SEVs from HIV-infected (HIV+, hereafter designated as HIV+ BEV, HIV+ SEV) and uninfected (HIV-, hereafter designated as HIV- BEV, HIV- SEV) donors were diluted 1:20,000 to 1:160,000 in filtered ultrapure water until the concentration was within the range for suitable measurements. This range corresponds to 50–300 average particles per frame when using ZetaView (Table S1). First, to ensure that the BEV and SEV preparations were suitable for ζ-potential measurements, we acquired 10 measurements of 5 cycles each per sample (Figure 1C). Numerous acquisitions are typical for testing stability of samples during measurement [45]; if ζ-potential values fluctuate during measurement, it is an indication that the sample is unstable i.e., dissociating, aggregating, sedimenting, or solubilizing [46]. We found that the variation in ζ-potential for all four samples (pool of *n* = 10, HIV−BEV, HIV−SEV, and HIV + BEV, HIV + SEV) was <5 mV (Figure 1C), indicating that the EVs (BEVs and SEVs) were stable during the measurement period. Second, we confirmed that BEV and SEV ζ-potential measurements are within the suitable concentration range by performing two measurements for each sample at the high- and low- ends of the concentration range recommended by the manufacturer. Although Debye–Hückel–Henry’s equation for ζ-potential determination does not have concentration as a variable [47], it is only applicable in a narrow range of concentration. Thus, drastic dilutions, which are required for NTA, may largely affect the quality of the measurements leading to potential erroneous interpretations [46,48]. Nonetheless, we observed no significant differences between the two dilutions (Figure 1D), indicating that BEV and SEV ζ-potential measurements were indeed concentration-independent. Third, ζ-potential depends on the protonation state at the surface of the measured particle and is thus largely affected by the pH of the solution. Therefore, we constantly monitored the pH of all measured samples which were diluted with ultrapure water. Buffered solution such as PBS was not used because the salts in PBS would build up through the tubing and connections of the NTA machine to skew measurements. The pH during measurements ranged between 5.6 and 5.9, similar to the measured pH of ultrapure water (pH = 5.8), which is slightly acidic because of the presence of carbonic acid that results from the dissolution of atmospheric carbon dioxide [49]. In summary, ζ-potential measurement of BEV and SEV is stable and the observed ζ-potential was not influenced by unspecific interactions. Finally, it is known that the size of vesicles is an important parameter that dictates the amount of lipids, surface glycan, and glycoprotein content of a vesicle, and potentially the vesicle surface charge. Consequently, we measured the particle sizes of BEVs and SEVs for all samples individually. The result shows no significant differences between BEVs and SEVs size irrespective of donor HIV status (Figure S1).

### 3.2. BEVs Bear Neutral Surface Compared to the More Anionic SEVs

**Figure 2:**
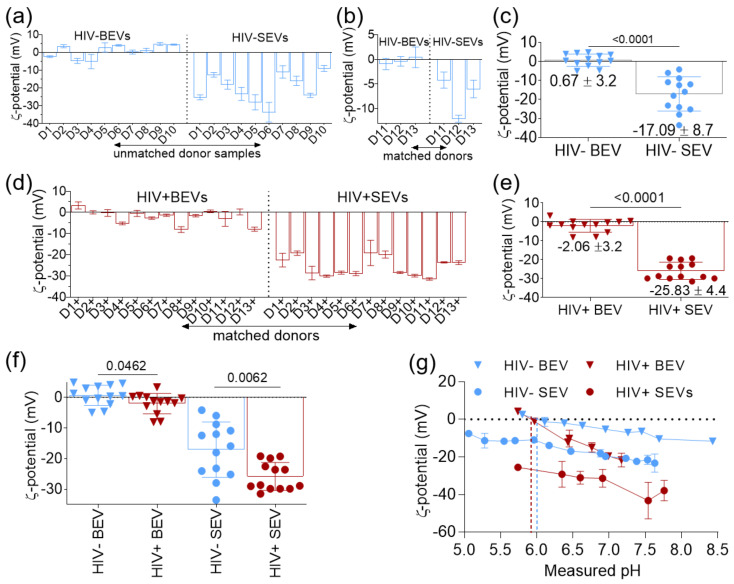
ζ-potential measurements of HIV- and HIV+ BEVs and SEVs. (**a**,**b**) Mean ζ-potential of quintuplet measurements of HIV- BEVs and HIV- SEVs from 10 unmatched donor samples (**a**) and 3 matched donor samples (**b**). Error bars correspond to S.D. (**c**) A graph showing the mean ζ-potential for all 13 HIV- BEV and HIV- SEV samples. Numbers below and above the data are, respectively, the mean of the means ± S.D and the *p*-value of an unpaired t-test with Welch’s correction. (**d**) Mean ζ-potential of quintuplet measurements of HIV+ BEVs and SEVs, from 13 matched donors. Error bars correspond to S.D. (**e**) Mean ζ-potential for all 13 HIV+ BEV and SEV samples. Numbers below the data are the mean of the means ± S.D. The number on top of the data corresponds to the *p*-value of an unpaired t-test with Welch correction. (**f**) Effects of HIV-1 on the ζ-potential of BEVs and SEVs. The numbers on top of the data represent the *p*-values of unpaired t-tests with Welch correction between the HIV- and HIV+ groups. (**g**) ζ-potential of pools (*n* = 10) of HIV- and HIV+ BEVs and SEVs as a function of pH. Blue and red dashed vertical lines correspond to the IEP of HIV- and HIV+ BEVs, respectively. Error bars are S.D. of quintuplet measurements.

ζ-potential of purified HIV- BEV and HIV- SEV from 10 unmatched HIV- donors was measured using ZetaView and quintuplet measurements for each sample were recorded. HIV- BEVs were neutral (−5–+4.8 mV), whereas HIV- SEV were negatively charged (−33.5–−8.8 mV) Figure 2a and Figure S2). To determine whether the difference in ζ-potential between BEV and SEV is universal or donor-dependent, we analyzed 3 additional HIV- BEV and HIV- SEV from matched donors (Figure 2b and Figure S2). Statistical analysis confirmed that HIV- BEV are generally neutral (mean ± SD = 0.67 ± 3.7) while HIV- SEV are negatively charged (mean ± SD = −17.09 ± 8.7), with a *p*-value of the difference <0.0001 (Figure 2c). This result suggests that the difference in ζ-potential between HIV- BEVs and HIV- SEVs may be due to membrane compositional differences between HIV- BEVs and HIV- SEVs.

### 3.3. HIV Infection Increases the Net Negative Charge on the Surface of BEVs and SEVs

To identify the effect of HIV infection on the electrostatic surface properties of BEV and SEV, purified HIV+ BEV and HIV+ SEV from 13 matched HIV infected ART-suppressed donors were analyzed for ζ-potential. HIV+ SEV were more negatively charged (−25.83 ± 4.4 mV) compared to HIV + BEV (−2.06 ± 3.2 mV) as expected (Figure 2d,e, Figure S2). Although BEVs remained generally neutral and SEVs negatively charged, a comparison of the ζ-potential values of HIV + EV with those from HIV- EVs indicated that HIV+ EVs tend to be slightly more negatively charged compared to HIV- EVs (Figure 2f). A two-tailed unpaired *t*-test between the two groups indicated that the observed difference was significant (*p* = 0.0462 and *p* = 0.0062, for BEVs and SEVs, respectively). To confirm this result, a pH dependence experiment was conducted in which a pool of EVs from each experimental group (*n* = 10) was pH-titrated and ζ-potential measured (Figure 2g). To assign numerical values to the surface charge of EVs, the isoelectric point (IEP) which corresponds to the pH at which the surface is neutral was determined graphically and is summarized in Table 1. The result confirmed the differences between HIV- and HIV+ groups for both BEVs and SEVs and indicated that HIV infection may indeed alter the EV membrane surface charge by increasing its net negative charge. The reasons for the differences between HIV- and HIV+ are unknown but may include HIV-induced (i) differences in EV composition or (ii) differences in the ratios of EV subtypes release. It is also possible that these differences may be related to the use of ART, since all HIV+ donors are virally suppressed by ART.

### 3.4. SEVs Internalization by Epithelial Cells is more Efficient Compared to BEVs and EVs Internalization Correlates with Their IEPs

To determine if HIV-induced surface charge modification of EVs impact their downstream function, we investigated the internalization of HIV- and HIV+ BEVs and SEVs. To this end, EVs from HIV+ and HIV- groups were separately pooled and labelled using an EV protein labelling dye. To confirm that labelling does not affect the EVs surface charge, ζ-potential and pH were measured before and after EV labeling (Table 2). Although the numbers varied, the labeling process produced no significant effect on ζ-potential; i.e., HIV- and HIV+ BEV were consistently in the slightly negative to neutral range (−10 to 0 mV), whereas HIV- and HIV+ SEV remained significantly more negative (−20 to −30 mV).

**Figure 3:**
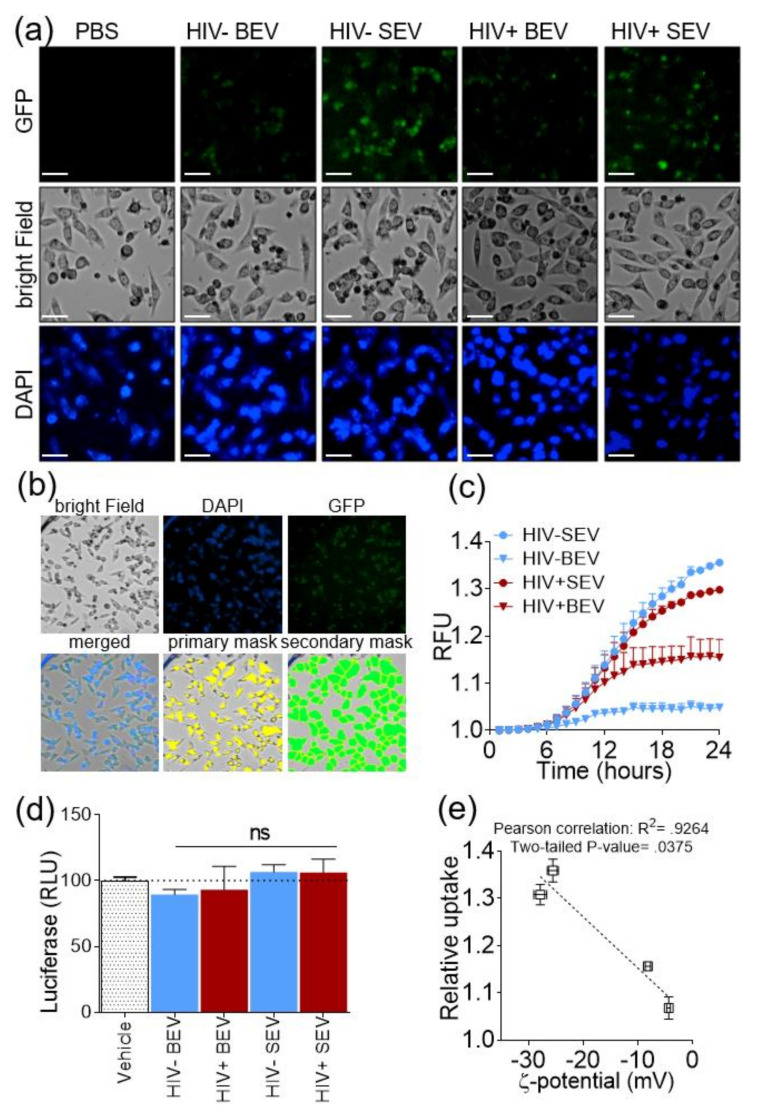
Internalization rate and efficiency of HIV- and HIV+ BEVs and SEVs by epithelial cells. (**a**) Representative images of TZM-bl cells 24 h post-internalization experiment. Scale bar is 50 µm. (**b**) Quantification strategy for EV internalization as described in Materials and Methods. (**c**) Kinetics of EV internalization. Error bars represent S.D. of 4 wells, each with stitched 4 fields of view. Experiment was repeated three times with similar results. (**d**) Luciferase assay post-internalization showing no LTR promoter activation beyond the basal level of cells, which was set to 100. “ns” indicates not significant based on one-way ANOVA, compared to PBS control. (**e**) Linear regression between relative internalization and ζ-potential of EVs.

Labeled HIV- and HIV+ BEVs and SEVs or labeled PBS control were mixed with DMEM media containing NucBlue stain and the mixture was added to TZM-bl cells. Kinetics of EV internalization by cells were monitored in Green-fluorescence protein (GFP), bright field, and DAPI channels every hour for 24 h using an automated scope. Representative images of the cellular internalization are shown in Figure 3a. During measurements, the parameters of the GFP acquisition channel were kept constant (LED = 5, acquisition time = 100 ms, and gain = 1), the GFP intensity per cell was determined using a secondary mask strategy set in the GFP channel whereas a primary mask was set in the DAPI channel (Figure 3b). The average intensity per cell in each treatment was normalized to time zero to correct for background and then normalized to that of labelled PBS control, which was set to 1, and the relative GFP intensity was plotted as a function of time (Figure 3c). Internalization efficiency was higher for SEV compared to BEV (Figure 3c). Rate of internalization plateaued earlier with BEVs (at hour 12) compared to SEVs that continued the upward trend up to end of the experiment at hour 24. To understand whether the difference in EV internalization is affected by the presence of infectious HIV particles in the EV preparation, we assessed HIV infectivity by measuring the relative luciferase intensity at the end of the internalization experiment, since TZM-bl cells contain LTR-luciferase reporter used for assessing HIV infectivity. No signal beyond basal luciferase levels was detected in the HIV- or the HIV+ EV treated cells (Figure 3d), indicating that the observed differences in EV internalization efficiency between the two groups do not result from the presence of free infectious HIV. This observation is not surprising because all HIV+ donors were ART-adherent and virally suppressed for 5+ years. Interestingly, we noticed that the relative internalization of BEVs and SEVs significantly correlate with the ζ-potential of the labeled EVs (Pearson correlation: R squared = 0.9264, two-tailed *p*-value = 0.0375) (Figure 3e). This correlation suggests that EV internalization may be influenced by the EV surface charge with more negatively charged vesicles being more efficiently internalized by cells.

### 3.5. SEVs Internalization Efficiency by Primary Immune Cells is Higher Compared to BEVs

**Figure 4:**
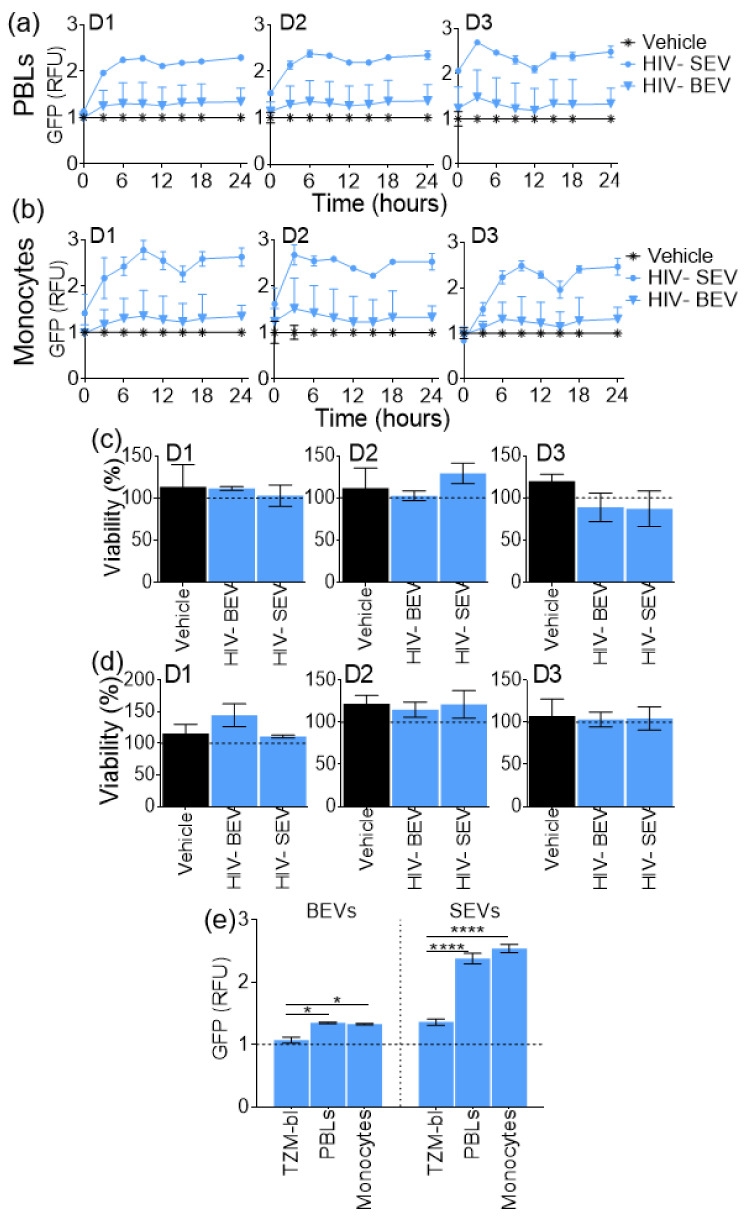
Internalization rate and efficiency of HIV- BEVs and SEVs by primary peripheral blood lymphocytes (PBLs) and monocytes. (**a**,**b**) Kinetics of EV internalization by (**a**) PBLs and (**b**) monocytes. Fluorescence intensity from the green-fluorescence protein (GFP) channel for each sample was normalized to that of labelled PBS which was set to 1. Error bars represent SD of 6 wells. Experiment was done three times with three different donors (D1-D3). (**c**,**d**) MTT viability assay post- internalization for (**c**) PBLs and (**d**) monocytes showing no difference in cell viability compared to labelled PBS which was set to 100. (**e**) Comparison of internalization efficiency amongst cell types at 24 h. 2-way ANOVA test (Sidak’s correction) was performed to determine the differences. Error bars indicate S.E.M. of 3 independent experiments. * *p* < 0.05; **** *p* < 0.0001.

TZM-bl cells studied in the previous section are epithelial cells from a cervical carcinoma cell line. They may thus be naturally more receptive to SEVs than BEVs. Hence, we sought to assess internalization efficiency of BEVs and SEVs in different cell lines, namely primary immune blood cells, PBLs, and monocytes. Labelled PBS control or labelled HIV- BEV or SEV were mixed with RPMI media at a concentration of 100 µg/mL and the mixture was added to primary PBLs or monocytes isolated from three healthy blood donors. The fluorescence intensity was recorded every 3 h for 24 h using an automated microscope. Total GFP intensity in each treatment was normalized to that of labeled PBS which was set to 1, and the relative GFP intensity was plotted as a function of time (Figure 4a,b). At the end of the internalization experiment, cell viability was measured with the MTT assay (Figure 4c,d). The results show that EVs entered the cells in the first three hours, with SEV being more efficiently internalized compared to BEVs both in PBLs (Figure 4a) and monocytes (Figure 4b), suggesting cell-type independency in EV internalization rate and efficiency. The MTT viability test showed no significant difference between the EV and PBS treated PBLs (Figure 4c) and monocytes (Figure 4d), suggesting that the observed differential internalization efficiency between SEVs and BEVs is independent of cell death. Comparison of internalization efficiency at 24 h amongst the three different cells types shows higher internalization of HIV- BEVs by immune cells as compared to TZM-bl (PBLs mean diff. = 0.278, Adj. *p*-value = 0.0270; monocytes mean diff 0.2591, Adj. *p*-value = 0.0449) (Figure 4e). Interestingly, SEV internalization at 24 h was also higher for PBLs and monocytes compared to TZM-bl (PBLs mean diff. = 1.016, Adj. *p*-value <0.0001; monocytes mean diff. = 1.177, Adj. *p*-value <0.0001). Taken together, these results suggest that immune cells internalize EVs to a greater degree than epithelial cells, irrespective of the EV type or source. This is not surprising, since immune cells are highly phagocytic. Additionally, EV internalization by target cells appears to be dictated by the charge on the surface of EVs.

### 3.6. Neuraminidase Treatment Decreases SEV Absolute ζ-Potential and Internalization Efficiency

Considering our findings that EV surface charge correlates with cellular internalization efficiency with more negatively charged EVs being more efficiently internalized, we hypothesized that SEVs, but not BEVs, are enriched with sialic acid residues conferring to SEVs their net negative charge. Indeed, EVs were found to bind with high affinity sialic-acid-binding lectins [31] and sialic acids have a low *pK*_a_ of 3.76 in aqueous solution [50]. EV pools from HIV- and HIV+ BEVs and SEVs were subjected to α2-3,6,8 neuraminidase treatment to hydrolyze α2-3, α2-6, and α2-8 linked sialic acid residues from surface glycoproteins. Three types of membrane molecules contain sialic acids: O-glycan, N-glycan and glycolipids such as sphingolipids (Figure 5a). It is important to note that neuraminidase treatment is minimally invasive and does not alter the glycocalyx coat nor the membrane proteins, hence preserving the EV membrane integrity [32]. As expected, sialic acid digestion decreased the net negative charge on HIV- and HIV+ SEVs but did not significantly change the ζ-potential of HIV- or HIV+ BEVs (Figure 5b). Intact and digested EVs were labeled with EV protein labelling dye and labelled EVs were used for internalization experiment in TZM-bl cells. Then, 24 h later, cells were imaged using an automated microscope at a fixed GFP intensity. Primary and secondary masks were set in the DAPI and GFP channels, respectively, allowing GFP intensity tracing per cell (Figure 5c). Analysis of the GFP data shows that SEVs were more significantly internalized as compared to BEVs (Figure 5d, filled bars). Upon neuraminidase treatment, internalization efficiency of both HIV- and HIV+ SEVs significantly decreased, whereas BEVs internalization was not affected (Figure 5d, two-group comparisons).

To gain insight into the differential response of BEVs and SEVs to neuraminidase, we performed a secondary analysis of our previously published Kaddour et al., proteomics dataset of BEVs and SEVs isolated from uninfected or HIV-infected ART-suppressed individuals [51]. The Kaddour et al., study identified 218 and 246 differentially present proteins (DPPs) in SEV as compared to BEVs in HIV- and HIV+ groups, respectively. A 2-way Venn analysis of the Kaddour dataset identified a total of 310 unique SEV-enriched DDPs, of which 154 proteins were common to HIV- and HIV+ SEVs, 64 and 92 proteins were enriched in HIV- and HIV+ SEVs, respectively (Figure 5e). To identify molecular pathways potentially perturbed by the DPPs, the 310 modulated proteins were mapped to KEGG database. Notably, the top two pathways with the highest enrichment ratio (Table 3) include the following terms: other glycan degradation and glycolysis/glycogenesis. This analysis revealed 21 glycocalyx-related SEV-enriched DPPs, including sialidase-1 (NEU1) known to be shed from sperm during capacitation [52] (Figure 5f).

## 4. Discussion

Here, we showed differential surface electrostatic properties of BEVs and SEVs, where SEV membranes bear a more negative net charge than BEV membranes. We also showed differences in BEV and SEV sialylation and desialylation, which correlated with their internalization efficiency by target cells. EVs are in general internalized by recipient cells through various mechanisms [54,55], including, phagocytosis [56], receptor-mediated internalization [57], and direct membrane fusion [57]. These EV internalization mechanisms require a minimal distance (a few nanometers) for an initial physical interaction to occur. These interactions are mainly orchestrated by electrostatic interactions and van der Waals forces, which may be a direct reflection of the surface composition and charge of the EVs and that of the recipient cells.

Considering our data, negative surface charges appear to drive EV internalization, albeit subtly for BEVs. This observation is counter intuitive and surprising because the cell membranes are also negatively charged, suggesting that a repulsion should rather occur. However, two plausible mechanisms may explain this perplexing phenomenon. First, EV internalization may be an active process in which EVs utilize locally charged microdomains on the cell surface membrane as a port of entry, rather than a passive entry through the lipid membrane per se. Indeed, cell membranes, although negatively charged in general, are asymmetric and contain non-uniform islands of specific lipids and macromolecules that exert bioactive roles, such as ceramide [58,59]. Furthermore, sphingosine, a cationic lipid, has been associated with negatively charged DNA cellular internalization through electrostatic interactions [60]. Thus, negatively charged EVs may cross the lipid membrane barrier through a similar mechanism. The second plausible mechanism is explained from a physical chemistry standpoint, where similarly charged macromolecules in dilute polar solutions tend to attract and self-assemble [61]. Although this phenomenon has been reported to occur in narrow size conditions of macroions (1–6 nm) [62], to form hollow blackberry-like supramolecular structures of tens to hundreds of nanometers [63], there is no reason to exclude its occurrence at the EV/recipient cell membrane interface. 

Mechanistically, the finding that SEV surface is sialic acid-rich is validated by the decrease in SEV surface negative charge upon neuraminidase-mediated desialylation. Interestingly, desialylated SEVs exhibited decreased internalization efficiency (Figure 5c,d), in line with recent studies [31,32,33,34,35]. Although our findings relate to body fluid EVs, similar observations have been made in cancer-associated cell line derived EVs. Akagi et al., used a microcapillary electrophoresis chip to demonstrate that six cancer cell lines secrete EVs that are more negatively charged than those secreted by their non-cancerous counterparts and that this negative charge is conferred by the presence of sialic acids [33,35]. Similarly, Shimoda et al., arrived at the same conclusion when they treated recipient cells and incoming exosomes with anti-CD33 (siglec-3) or soluble sialic acid, both of which impeded incoming EV internalization [31]. Conversely, Williams et al., [32] found that neuraminidase treatment in general enhanced EV internalization by a panel of 28 human cell lines, but with stark differences that the authors correlated to differential EV biodistribution upon neuraminidase treatment in vivo [34]. The authors argued that sialic acid digestion, by decreasing EV negative surface charge and decreasing steric hindrance, may favor EV-recipient cell attraction. This explanation is based on the fact that some cell membranes may be more negatively charged compared to desialylated EVs; in which case, more electrostatic interactions would drive non-specific passive EV internalization. However, the steric hindrance explanation may not apply because the molecular weight of sialic acid (309 Daltons) is insignificant compared to that of EVs (100–150 million Daltons, [64]). Instead, as evidenced in our study and elsewhere [33,35], the presence of sialic acid positively correlates with cellular internalization, pointing to the possibility of specific and active internalization mechanism. Nonetheless, it is likely that the process of EV internalization is not exclusively sialic acid dependent but may also involve the repertoire of other membrane proteins or molecules on the surface of EVs and on recipient cells. The process may also involve other heterotypic interactions, including glycocalyx, receptor, and/or glycolipid dependency—all of which contribute to different extents to the EV-mediated cellular communication [65]. These findings altogether highlight the role of sialic acid in particular, and of glycocalyx in general, in EV internalization, and underscore the complexity of the mechanisms of cellular internalization.

It is known that both blood and seminal plasma contain sialic acid and the levels are higher in semen compared to blood [66]. However, this is the first study to show that the sialic acid signature of body fluids is preserved in their EVs. Because sialic acid mark terminal glycans, especially galactose residues [67] (Figure 5a), it is thus implied that SEVs possess a thicker glycocalyx compared to BEVs. Enrichment of SEVs with molecules involved in pathways related to other glycan degradation and glycolysis/gluconeogenesis, including Neu1 (Figure 5f, Table 3) corroborates this observation. Indeed, Neu1 is one of the enzymes that modulate spermatozoa sialome via cleavage of sialic acid molecules from sialoglycoconjugates during capacitation, thus enhancing fertilization [52]. This is important because, although sialic acid-rich glycocalyx coats on spermatozoa correlate positively with protection of spermatozoa from phagocytosis, it negatively correlates with the binding of spermatozoa to the zona pellucida of the ovum [68,69,70]. While the precise role of SEV Neu1 against SEV sialic acid is unknown, it is likely that SEV Neu1 may be involved in controlled enzymatic loss of sialic acid from the surface of spermatozoa. Such endogenous desialylation may allow uncapped desialylated glycans to engage lectins in the female reproductive tract. This process may promote clustering of the spermatozoa surface glycoconjugates and signaling, in a process similar to the interaction of T-cell and dendritic cell in virological/immunological synapses. Having identified SEV Neu1 and ability of Neu1 to alter the surface electrical charge of SEVs, these findings opens the way to studies of SEV sialic acid and Neu1 modulation of spermatozoa capacitation, or the effect of SEV Neu1 in unmasking key receptors for sialylated ligands on the egg zona pellucida.

Another implication of our findings is that activated immune cells internalized SEVs more than BEVs (Figure 4e). This increased SEV internalization into blood-derived immune cell types may have clinical relevance during HIV infection. SEVs present in seminal plasma maybe transported across mucosal membranes to enter the blood stream, and then internalized by blood-resident immune cells such as T-cells and monocytes. Thus, studies designed to identify semen resident EVs in blood are underway. While we have demonstrated that SEV and BEV from HIV+ do not contain HIV particles, it is established that these EVs can contain other viral cargos, such as the viral Nef protein [71,72] and various microRNAs [73,74]. In addition, these EVs are known to contain molecules involved in host antiviral and inflammatory responses [7]. An interesting continuation of this work would be to investigate HIV EV cargos in SEV and BEVs from HIV infected patients. It is known that differences in EV cargo, composition, and function exist and that these differences are exemplified in HIV infection studies. For instance, BEVs have little or no effect in HIV infection while SEVs [37,38], breast milk Evs [75], and vaginal fluid EVs [76] inhibit HIV infection. These stark differences in the functions of EVs are even more remarkable when autologous EVs were used [38,51]. Indeed, considering our previous findings that SEVs, but not BEVs—unless they carry antiretroviral drugs [38]—inhibit HIV [36,37,40,41], independent of immune cell activation [39], it is possible that sialic acid content of SEVs may play critical role in HIV pathogenesis. One class of sialic acid receptors are sialic acid-binding immunoglobulin-like lectins (Siglecs), which upon sialic acid ligation act to dampen host immune responses and set appropriate thresholds of immune activation [24]. HIV, like many pathogens, takes advantage of this mechanism; with terminal sialic acids on viral envelope gp120 proteins binding Siglec-7 on host monocyte/macrophage, natural killer, and CD4^+^ T cells [77,78]. Viral sialic acids also interact with Siglec-1 on host monocytes/macrophage and dendritic cells [79,80], and Siglec-1 mediates macrophage to CD4^+^ T cell HIV transmission [81]. All of these viral sialic acid•siglec interactions promote virus binding, subsequent virus entry, and infection [79,82]. Thus, it is possible that sialic acid rich SEVs saturate host cell siglecs and competitively reduce available siglecs for HIV envelope sialic acid binding. SEVs may also dimerize with HIV particles via sialic acid, thereby preventing HIV binding via viral sialic acid masking and/or steric hindrance of the HIV•EV dimer; similar to what has been proposed when recipient cells release exosomes that pair with incoming exosomes and modulate signaling mediated by the incoming exosomes [83]. On the other hand, it was shown that desialylation of incoming virions resulted in enhanced HIV [84] and HRSV (human respiratory syncytial virus) [85] infection. In this scenario, sialic acids act as infection attenuators. However, it has been reported that desialylation of recipient cells did not enhanced HRSV infection [85], but removal of target cell sialic acids enhanced infectivity of HIV, as well as HIV-pseudotypes bearing murine leukemia virus and vesicular stomatitis virus proteins [84]. However, these potential interactions require further study in our system.

To our knowledge, this is the first study to report on HIV-induced alteration in body fluid EV surface charge. Our observation that HIV infection increased the negative surface charge on BEVs and SEVs (Figure 3c) is novel, and in line with the notion that HIV reprograms the epigenome and proteome of host cells [86,87,88,89,90,91] and EVs [51,92]. The reason for the differences in surface charge between HIV+ and HIV- BEVs and SEVs is unknown. It is tempting to speculate that despite being significantly virally suppressed (<32 genome equivalents/mL), low levels of HIV proteins may be associated with EV membranes. It is also plausible that HIV+ BEVs and SEVs may be associated with negatively charged lipids, such as, the microbial products lipopolysaccharides (LPS) or LPS-binding protein (LBP) that have been shown to infiltrate the blood and tissues of SIV and HIV+ rhesus macaques and humans [93,94,95]. Additional studies with increased sample size are needed to examine the relationship between EV surface charge and microbial products contents in people living with HIV (PLWH). Furthermore, what is warranted is the use of purer EV preparations such as those isolated by particle purification liquid chromatography (PPLC) [96], since the EVs described in this study were isolated using the precipitation method. Other questions that remain unanswered include (i) why the ζ-potential of BEV differ from SEV? (ii) What is ubiquitous on the surface of HIV+ BEVs and SEVs that is not on HIV- EVs?

Finally, in addition to its role in EV internalization, surface electrostatic properties may be important in the application of EVs in drug delivery. Over the past several decades, a wide array of synthetic drug delivery modalities have been developed and brought to market. However, overall success rates have been low due to cytotoxicity, immunogenicity, and inefficient cellular uptake. Given their physiological origin, EVs have been shown to exhibit minimal cytotoxicity and immunogenicity in vivo [97,98]. In addition, EVs are intrinsically able to interact with plasma membranes of recipient cells through a variety of ligand–receptor interactions and have been shown to efficiently cross these barriers to administer a variety of therapeutic nucleic acid and protein cargos to induce functional changes in target cells in multiple neurodegeneration and cancerous disease states [99,100,101]. In the context of cancer, however, some tumor cells are capable of modulating their internalization capacities of anti-cancer drugs by acidifying their local microenvironment. This also increases release of tumor cell EVs, which have been shown to be selectively enriched with chemotherapy drugs; culminating as a net efflux of such drugs [102]. One group demonstrated that conjugation of exogenous peptide sequences to mesenchymal stromal cell EVs was able to significantly increase uptake in ischemic brains to deliver therapeutic cargo [103]. This peptide had high affinity for avb3 integrins, which are highly expressed by vascular endothelial cells after ischemia. Thus, understanding vesicular components that regulate cellular uptake, such as those demonstrated in this current manuscript, may have a positive impact on the drug delivery field. Furthermore, direct pharmacological manipulation of EV ζ-potential has also been shown to generate promising anti-cancer effects [104]. These examples, along with the results presented here, highlight the critical roles of the surface electrical charges of EV. The wide-spanning implications of EV internalization in host health and disease, as well as their potential in therapeutics [105,106,107,108], underscores the importance of the findings of this study and the value of interdisciplinary approaches in research.

## Figures and Tables

**Table 1:** IEP of BEVs and SEVs. ^1.^

EV	IEP
HIV − BEV	~6
HIV − SEV	<5.5
HIV + BEV	~5.9
HIV + SEV	<5.5

^1^ Isoelectric point, which is also known as point of zero charge (PZC) and corresponds to the pH at which ζ-potential = 0 mV, was graphically determined from panel (d) of Figure 3.

**Table 2:** Effects of exosome labelling on the ζ-potential of BEVs and SEVs.

	Before Labelling		After Labelling	
	Measured pH	ζ-Potential	Measured pH	ζ-Potential
HIV- BEV	5.74 ± 0.14 ^1^	−0.5 ± 1.1	5.71 ± 0.11	−4.4 ± 0.3
HIV- SEV	5.83 ± 0.12	−23.3 ± 0.7	5.74 ± 0.12	−25.6 ± 1
HIV+ BEV	5.78 ± 0.14	−5.5 ± 0.6	5.67 ± 0.14	−8.2 ± 0.5
HIV+ SEV	5.82 ± 0.13	−28.1 ± 2.6	5.64 ± 0.14	−27.9 ± 1.2

^1^ Experiment was repeated three times, each with triplicate measurements. Results are presented as mean ± S.E.M.

**Table 3:** Top-10 KEGG pathways of all 310 significantly enriched proteins in SEVs as compared to BEVs in HIV- and HIV+ groups.

GeneSet	Description	Size	Overlap	Expect	Enrichment Ratio	*p*-Value	FDR ^£^
**hsa00511**	O**ther glycan degradation**	**18**	6 *	**0.500068766**	**11.99834983**	**6.00 × 10^−6^**	**2.17 × 10^−4^**
**hsa00010**	**Glycolysis/Gluconeogenesis**	**68**	**15 ^#^**	**1.889148673**	**7.94008445**	**3.27 × 10^−10^**	**5.34 × 10^−8^**
hsa04142	Lysosome	123	23	3.41713657	6.730781615	2.06 × 10^−13^	6.71 × 10^−11^
hsa04612	Antigen processing and presentation	77	13	2.139183056	6.07708628	1.54 × 10^−7^	1.00 × 10^−5^
hsa01200	Carbon metabolism	116	16	3.222665383	4.964834414	1.05 × 10^−7^	8.53 × 10^−6^
hsa05322	Systemic lupus erythematosus	133	15	3.694952551	4.059592049	3.64 × 10^−6^	1.70 × 10^−4^
hsa05203	Viral carcinogenesis	201	22	5.584101224	3.939756662	3.02 × 10^−8^	3.29 × 10^−6^
hsa04141	Protein processing in endoplasmic reticulum	165	17	4.583963691	3.708580858	2.85 × 10^−6^	1.55 × 10^−4^
hsa05034	Alcoholism	180	17	5.000687663	3.399532453	9.32 × 10^−6^	3.04 × 10^−4^
hsa01100	Metabolic pathways	1305	62	36.25498556	1.710109632	5.52 × 10^−6^	2.17 × 10^−4^

* MANBA, FUCA1, HEXA, HEXB, GLB1, and NEU1; ^#^ LDHA, PGK1, GAPDH, ENO1, GPI, LDHB, PGK2, LDHC, FBP1, ALDOC, ADH5, AKR1A1, PKM, ALDH9A1, and TPI1; ^£^ False Discovery Rate. Bold = Top-2 pathways based on the enrichment ratio as determined by the Webgestalt analysis using the default parameters (http://www.webgestalt.org/ accessed on 13 June 2020), of which the overlap genes were used for Figure 5f.

## Supplementary Materials

The following are available online at https://www.mdpi.com/1999-4915/12/10/1117/s1, Table S1: Parameters of the Zeta potential measurements with Zeta-View, Figure S1: Particle size measurements of HIV- and HIV+ BEVs and SEVs, Figure S2: Representative plots of ζ -potential measurements for HIV- BEVs and SEVs, Figure S3: Representative plots of ζ -potential measurements for HIV+ BEVs and SEVs.
