# Supplementary material for: Electrostatic Surface Properties of Blood and Semen Extracellular Vesicles: Implications of Sialylation and HIV-Induced Changes on EV Internalization
Source: Viruses. 2020 Oct 1;12(10):1117. doi: 10.3390/v12101117 (PMC7601085; doi:10.3390/v12101117)
Supplement: Supplementary file 1 [file viruses-12-01117-s001.pdf]

Electrostatic surface properties of blood and semen extracellular vesicles: Implications of sialylation and HIV-induced changes on EV internalization

Hussein Kaddour [1], Tyler D. Panzner [1], Jennifer L. Welch [2,3,4], Nadia Shouman [1], Mahesh Mohan[5], Jack T. Stapleton [2,3,4], and Chioma M. Okeoma [1,*]

[1] Department of Pharmacology, Stony Brook University Renaissance School of Medicine, Stony Brook, NY 11794-8651, USA
[2] Department of Microbiology and Immunology, Carver College of Medicine, University of Iowa, Iowa City, Iowa, USA
[3] Medical Service, Iowa City Veterans Affairs Medical Center, Iowa City, IA 52246-2209, USA
[4] Department of Internal Medicine, Carver College of Medicine, University of Iowa, Iowa City, IA 52242-1109, USA
[5] Host Pathogen Interaction Program, Southwest National Primate Research Center, Texas Biomedical Research Institute, San Antonio, Texas, 78227, USA; mmohan@txbiomed.org
* Correspondence: chioma.okeoma@stonybrook.edu; Tel.: +1-631-444-3014

Supplementary file

This file contains:

- Table S1. Parameters of the ζ -potential measurements with ZetaView.
- Figure S1. Particle size measurements of HIV- and HIV+ BEVs and SEVs.
- Figure S2. Representative plots of ζ -potential measurements for HIV- BEVs and SEVs.
- Figure S3. Representative plots of ζ -potential measurements for HIV+ BEVs and SEVs.

Table S1. Parameters of the ζ -potential measurements with Zeta-View.

	Donor ID	EV	Dilution factor	particles per frame[¥]	number of traced particles[£]
HIV-	1	BEV	160,000	151 ± 5	2128 ± 56
		SEV	160,000	123 ± 3	2895 ± 65
	2	BEV	200,000	50 ± 14	1337 ± 335
		SEV	200,000	44 ± 5	753 ± 247
	3	BEV	160,000	49 ± 9	1687 ± 198
		SEV	40,000	56 ± 14	1943 ± 380
	4	BEV	80,000	73 ± 19	1492 ± 202
		SEV	80,000	49 ± 24	1559 ± 173
	5	BEV	40,000	180 ± 97	1824 ± 384
		SEV	80,000	149 ± 66	3576 ± 620
	6	BEV	80,000	169 ± 8	4241 ± 215
		SEV	80,000	97 ± 22	3129 ± 393
	7	BEV	160,000	66 ± 49	2015 ± 284
		SEV	160,000	166 ± 65	3487 ± 598
	8	BEV	160,000	100 ± 31	3489 ± 423
		SEV	80,000	127 ± 80	2668 ± 533
	9	BEV	20,000	149 ± 9	3634 ± 72
		SEV	160,000	93 ±	3117 ± 304
	10	BEV	40,000	146 ±	3829 ± 360
		SEV	80,000	106 ±	2118 ± 318
	**11**	**BEV**	80,000	49 ± 37	2546 ± 642
		**SEV**	40,000	120 ± 14	3118 ± 452
	**12**	**BEV**	200,000	175 ± 9	4602 ± 107
		**SEV**	40,000	94 ± 24	3279 ± 276
	**13**	**BEV**	80,000	45 ± 6	1730 ± 318
		**SEV**	40,000	130 ± 11	3695 ± 259
HIV+	**1+**	**BEV**	40,000	241 ± 30	4874 ± 470
		**SEV**	80,000	169 ± 7	4490 ± 100
	**2+**	**BEV**	40,000	117 ± 23	3910 ± 334
		**SEV**	40,000	241 ± 18	4654 ± 420
	**3+**	**BEV**	40,000	98 ± 10	3231 ± 240
		**SEV**	40,000	98 ± 19	3430 ± 251
	**4+**	**BEV**	160,000	66 ± 10	1382 ± 240
		**SEV**	40,000	41 ± 2	1378 ± 115
	**5+**	**BEV**	20,000	112 ± 27	2761 ± 360
		**SEV**	20,000	48 ± 1	1305 ± 119
	**6+**	**BEV**	20,000	62 ± 17	2057 ± 216
		**SEV**	20,000	41 ± 1	1093 ± 86
	**7+**	**BEV**	40,000	70 ± 9	2661 ± 834
		**SEV**	80,000	70 ± 32	1986 ± 413
	**8+**	**BEV**	40,000	49 ± 3	1757 ± 54
		**SEV**	40,000	80 ± 2	2389 ± 3
	**9+**	**BEV**	40,000	80 ± 13	2007 ± 236
		**SEV**	40,000	125 ± 10	1021 ± 105
	**10+**	**BEV**	80,000	80 ± 7	2883 ± 85
		**SEV**	160,000	75 ± 12	2684 ± 138
	**11+**	**BEV**	80,000	43 ± 25	1459 ± 960
		**SEV**	80,000	55 ± 4	2177 ± 164
	**12+**	**BEV**	80,000	72 ± 28	1796 ± 94
		**SEV**	80,000	39 ± 6	1409 ± 845
	**13+**	**BEV**	160,000	89 ± 10	2714 ± 74
		**SEV**	80,000	42 ± 19	1752 ± 100

[¥] this parameter shows if samples were diluted to the right range where NTA is most accurate. Good PPF range is 40 – 250

[£] this parameter shows the total number of particles analyzed during a measurement

[*] Errors represent standard deviation of 3 to 10 measurements per sample. Higher number of measurements was taken to test the stability of certain samples, which turned to be stable

Bold = samples from matched donors

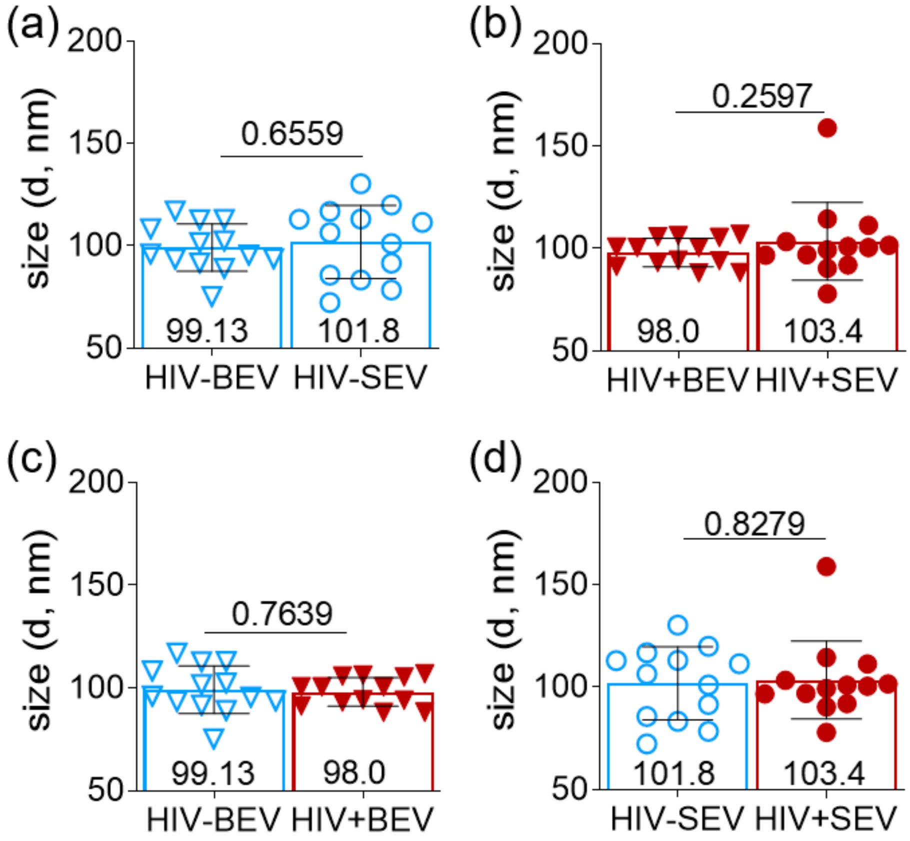

Figure S1. Particle size measurements of HIV- and HIV+ BEVs and SEVs. Purified EVs were diluted to the appropriate concentration with ultrapure H_2O (1:320,000 – 1:20,000) and size measurements (1 cycle at 11 positions) were undertaken using ZetaView PMX 110 (Particle Metrix, Mebane, NC, USA) and the corresponding software (ZetaView v8.5.5.2). Post-acquisition parameters were fixed as follows: min brightness, 20; min size, 10; max size, 4000; and tracelength, 15. Camera control settings were as follows: sensitivity, 90; frame rate, 30; and shutter, 70. (a) HIV- BEVs and SEVs. n=13, 10 unmatched- and 3 matched-donor samples. The number above the bars represents the *p*-value of an unpaired t-test with Welch's correction. (b) HIV+ BEVs and SEVs. n=13 matched-donor samples. The number above the bars represents the p-value of a paired and parametric t-test. (c,d) Comparison of HIV- and HIV+ BEVs (c) and SEVs (d). Numbers above bars represent *p*-values of unpaired t-tests with Welch's correction. For all graphs, each data point represents mean size of triplicate measurements, numbers inside bars correspond to the mean of the means, and error bars correspond to S.D.

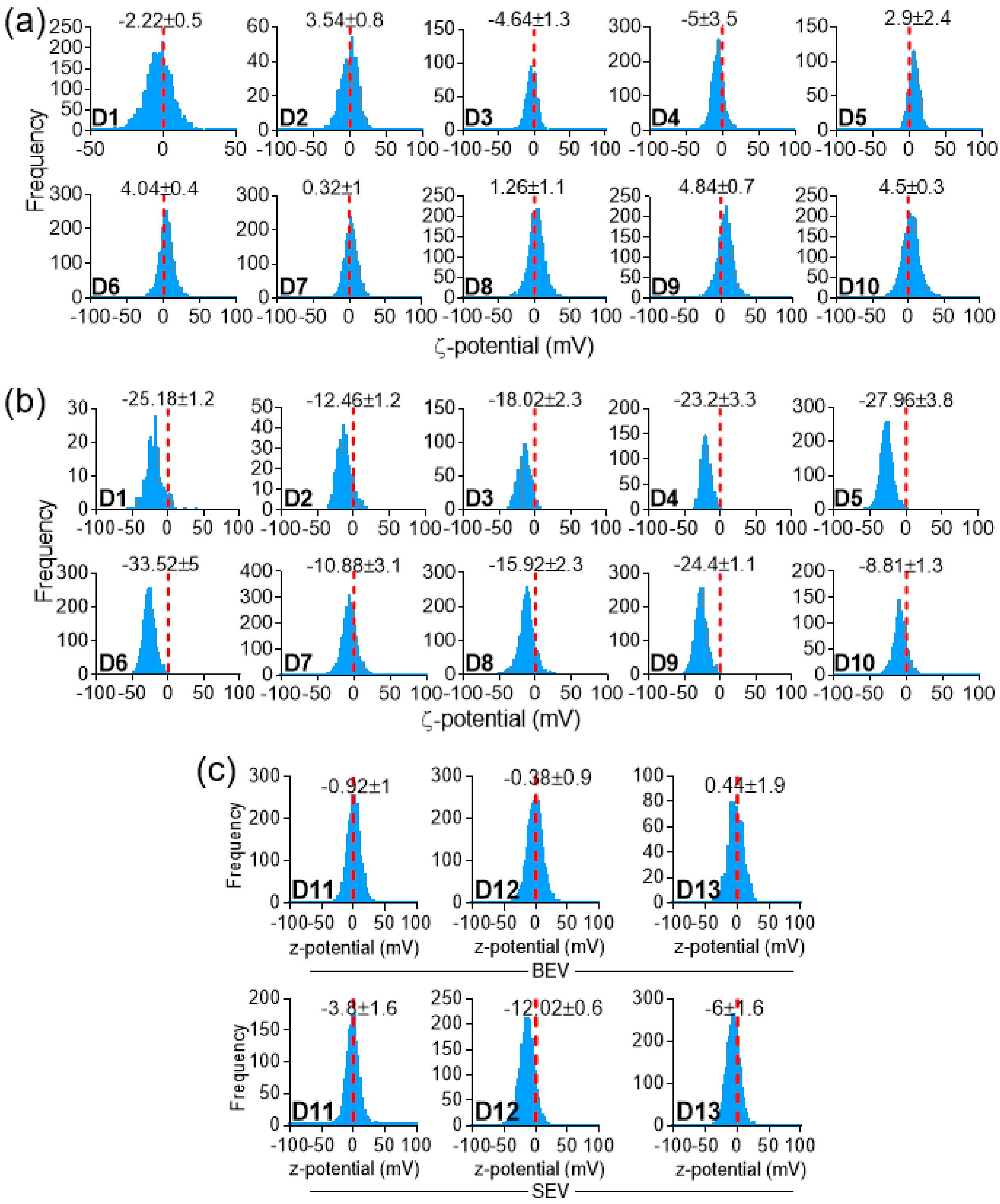

Figure S2. Representative plots of z-potential measurements for HIV- samples. (a) BEVs and (b) SEVs, from 10 non-autologous donors (D1-D10). (c) BEVs and SEVs from 3 matched donors (D11-13). Red dashed vertical line delimits 0 mV. Numbers inside graphs are mean values ± S.D. of quintuplet measurements.

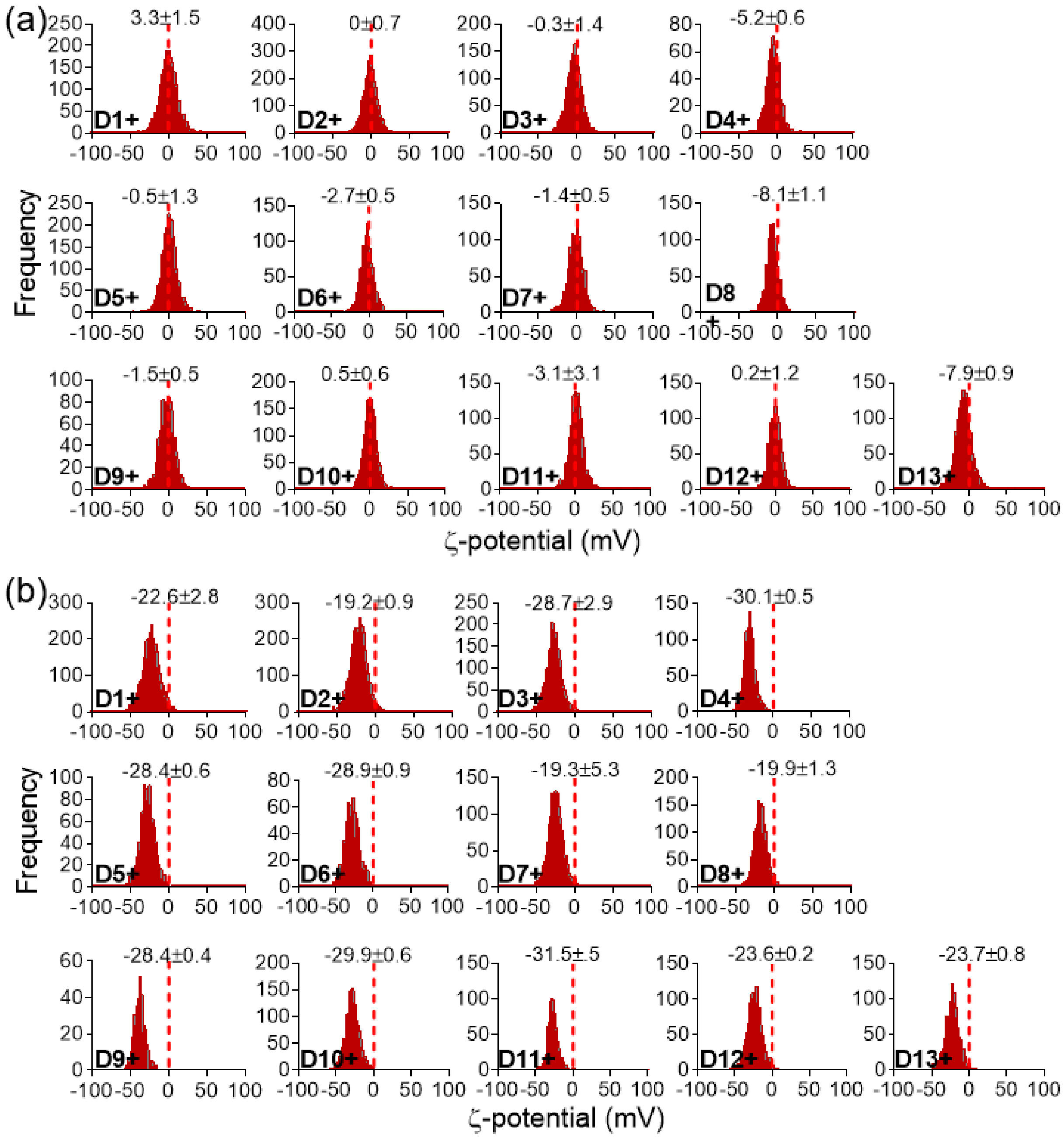

Figure S3. Representative plots of z-potential measurements for HIV+ samples. (a) BEVs and (b) SEVs from 13 autologous donors (D1-D13). Red dashed vertical line delimits 0 mV. Numbers inside graphs are mean values ± S.D. of quintuplet measurements.
